# Brainstem atrophy is linked to extrapyramidal symptoms in frontotemporal dementia

**DOI:** 10.1007/s00415-022-11095-x

**Published:** 2022-04-04

**Authors:** Sami Heikkinen, Antti Cajanus, Kasper Katisko, Päivi Hartikainen, Ritva Vanninen, Annakaisa Haapasalo, Johanna Krüger, Anne M. Remes, Eino Solje

**Affiliations:** 1 Institute of Clinical Medicine - Neurology, University of Eastern Finland, P.O. Box 1627 (Yliopistonranta 1C), 70211 Kuopio, Finland; 2 Neuro Center, Kuopio University Hospital, Kuopio, Finland; 3 Department of Radiology, Kuopio University Hospital, Kuopio, Finland; 4 Institute of Clinical Medicine - Radiology, University of Eastern Finland, Kuopio, Finland; 5 A.I. Virtanen Institute for Molecular Sciences, University of Eastern Finland, Kuopio, Finland; 6 Research Unit of Clinical Neuroscience, Neurology, University of Oulu, Oulu, Finland; 7 MRC, Oulu University Hospital, Oulu, Finland

**Keywords:** Frontotemporal dementia, Extrapyramidal symptoms, Progressive supranuclear palsy, Corticobasal degeneration, Imaging

## Abstract

Extrapyramidal (EP) symptoms are a known feature in a subpopulation of patients with behavioral variant frontotemporal dementia (bvFTD). Concomitant EP symptoms with FTD-like neuropsychiatric symptoms are also core features in progressive supranuclear palsy (PSP) and corticobasal degeneration (CBD). This complicates the early diagnosis of these disorders. Our retrospective register study aimed to discover imaging (MRI and FDG-PET) biomarkers to differentiate PSP, CBD, and bvFTD patients with extrapyramidal symptoms (EP +) from bvFTD patients without EP symptoms (EP-). The records of 2751 patients were screened for the diagnoses and presence of EP symptoms. A total of 222 patients were submitted to imaging analysis and applicable imaging data were recovered from 139 patients. Neuroimaging data were analyzed using Freesurfer software. In the whole cohort, EP + patients showed lower volumes of gray matter compared to EP- patients in the putamen (*p* = 0.002), bilateral globus pallidum (*p* = 0.002, *p* = 0.042), ventral diencephalon (*p* = 0.002) and brain stem (*p* < 0.001). In the bvFTD subgroup, there was volumetric difference between EP + and EP− patients in the brain stem. FDG-PET scans in the bvFTD patient subgroup showed that EP + patients had comparative hypometabolism of the superior cerebellar peduncle (SCP) and the frontal lobes. We discovered that EP symptoms are linked to brainstem atrophy in bvFTD patients and the whole cohort. Also, evident hypometabolism in the SCP of bvFTD EP + patients was detected as compared to bvFTD EP− patients. This could indicate that the EP symptoms in these diseases have a more caudal origin in the brainstem than in Parkinson’s disease.

## Introduction

Parkinsonism is a combination of certain extrapyramidal (EP) movement symptoms, usually defined by bradykinesia combined with either resting tremor or rigidity. Atypical parkinsonisms, such as progressive supranuclear palsy (PSP) and corticobasal degeneration (CBD), are rare diseases that share common clinical and neuropathological features (tauopathies) with frontotemporal dementia (FTD) [1]. The most prevalent of the clinical FTD spectrum diseases is behavioral variant frontotemporal dementia (bvFTD) [2]. BvFTD is commonly characterized as a cognitive and behavioral disease, but association between EP symptoms and bvFTD has also been known for decades. Akinesia, rigidity, and tremor were introduced as supportive diagnostic features in the 1998 diagnostic criteria of FTD [3]. These features were removed from the 2011 diagnostic criteria [2], but especially atypical parkinsonism remains a common presentation in bvFTD patients. In different FTD cohorts, parkinsonism has been observed in approximately 23% of the patients [4, 5]. The hexanucleotide repeat expansion in the *C9orf72* gene is the most common cause of familial FTD, and parkinsonism has been reported in 25–35% of patients carrying the expansion [6, 7]. Correlation of frontal and anterior temporal cortical atrophy with FTD is well established [8–12], but the structural and functional correlates of the EP symptoms remain to be explored in these patients.

In this retrospective register study, we aimed to identify imaging biomarkers using magnetic resonance imaging (MRI) and fluorodeoxyglucose-positron emission tomography (FDG-PET) that differentiate PSP, CBD, and bvFTD patients presenting EP symptoms from the bvFTD patients without EP symptoms.

## Materials and methods

A total of 2751 patients were identified between January 2010 and August 2020 from Kuopio University Hospital patient archive with a wide search of congruous ICD-10 codes, including codes for Parkinson’s disease and other neurogenerative diseases (G31, F02-F03, G23, G12.2, G25, G20, F04). The patient records were screened by an experienced physician specialized in neurodegenerative diseases to further specify the relevant diagnosis (bvFTD, PSP or CBD). A total of 222 patients with PSP (*n* = 50), CBD (*n* = 23), primary progressive aphasia (PPA) (*n* = 12), or bvFTD and FTD-ALS (*n* = 137) were identified. The presence of EP symptoms was defined if at least two of the following symptoms were present: rest tremor, bradykinesia, rigidity, prominent hypomimia, postural instability, and loss of automated movements. Based on these criteria, the 222 patients were finally grouped either as EP + (patients with EP symptoms) or EP- (patients without EP symptoms). Acceptable imaging data (MRI and/or FDG-PET) was available from 139 patients. The rest of the patients were excluded due to inadequate sequences, issues in image analysis, or obvious gross pathologies causing symptoms (e.g., brain tumors or chronic infarctions). The MRI and FDG-PET examinations were performed in the early diagnostic phase and the imaging data were collected retrospectively from the picture archiving and communication system. The study was approved by the Ethics Committee of the Hospital District of Northern Savo.

### MRI acquisition and analysis

Due to the retrospective nature of our study, the scans were obtained with different scanners (Phillips Achieva TX, Siemens Avanto, or Siemens Aera) and variable parameters. The field strength varied from 1.5T (*n* = 76) to 3.0T (*n* = 52). An appropriate 3D T1-weighted MRI scan acquired in the coronal or sagittal plane was available for 128 patients. Slice thickness varied from 0. to 1 mm.

The scans were preprocessed and analyzed using Freesurfer version 7.1.1 image analysis suite. The precise pipeline is demonstrated at http://surfer.nmr.mgh.harvard.edu. In short, processing included motion correction and averaging of multiple volumetric T1-weighted images, removal of non-brain tissue using a hybrid watershed/surface deformation procedure, automated Talairach transformation, segmentation of the subcortical white matter (WM) and deep gray matter (GM) volumetric structures, intensity normalization, tessellation of the GM/WM boundary, automated topology correction, and surface deformation following intensity gradients to optimally place the gray/white and gray/cerebrospinal fluid borders at the location where the greatest shift in intensity defines the transition to the other tissue class. Cortices were parcellated with Desikan atlas, and thicknesses of cerebral lobes were merged as defined in the original publication. Subcortical segmentation was performed using probabilistic atlas. Freesurfer morphometric procedures have been demonstrated to show good test–retest reliability across scanner manufacturers and field strengths [13, 14]; thus, we permitted the use of heterogeneous field strengths.

### ^18^Fluorodeoxyglucose PET acquisition and analysis

^18^Fluorodeoxyglucose PET scans were analyzed from patient archives retrospectively. FDG-PET scanning was performed at the Kuopio University Hospital. Subjects were scanned supine in a quiet room, instructed to remain awake with eyes open or closed. An injection of 200 MBq of [18F]-2-fluoro-2-D-glucose IV was used. The scan was commenced 60 min after tracer injection, and the duration of the scan was 15 min. FDG-PET scans were available for 64 patients.

PET scans were preprocessed and analyzed with SPM12 (Wellcome Trust Centre for Neuroimaging, London, UK; http://www.fil.ion.ucl.ac.uk/spm) software, running on Matlab 2019b (The Mathworks, MA, USA). MRI scans were co-registered to PET scans to minimize normalization errors, and then spatially normalized to the T1 MNI152 (Montreal Neurological Institute) template with a non-linear registration. The PET scans were corrected for partial volume effects using a three tissue compartmental algorithm (Müller–Gärtner method) with PETPVE12 toolbox [15]. Then, the FDG-PET images were normalized to the average count of cerebellar gray matter using an algorithm implemented in PETPVE12 toolbox [16]. Finally, images were smoothed with full width half maximum 8 mm Gaussian kernel to deal with subtle anatomical variation.

### Statistical methods

Statistical analyses were performed with IBM SPSS Statistic 27. Student’s t-test and Pearson's Chi-squared tests were used to assess differences across EP + and EP- groups regarding age and gender distributions.

General linear model, with age at scan as covariate, was used to compare groups as for the cortical thickness and subcortical volumes. The results were corrected for intracranial volume by a simple division. The results were not corrected for multiple comparisons, however only *p*-values < 0.01 were considered statistically significant. Considering FDG-PET, after preprocessing steps, scans were analyzed using a linear model with age at scan as a covariate. Regional hypometabolism was tested by a linear contrast (EP + vs. EP-) with a statistical threshold of *p* < 0.05 with a family-wise error (FWE) correction for multiple comparisons at the voxel-level, with minimal cluster size at *k* = 80. Since the FWE-corrected results yielded scarce statistically significant results, we also present uncorrected exploratory results that showed *p* value < 0.001.

### Data availability

The data that support the findings of this study are available from the corresponding author (E.S.) upon reasonable request.

## Results

### Demographic data

A total of 222 patients with a relevant diagnosis were included in the initial study cohort. Of the 222 patients, 45.9% (*n* = 102) met the EP + symptom criteria. From the subgroup of 139 patients with applicable imaging data available, EP + symptoms were present in 66 cases (47.5%). Divided into different clinical presentations, applicable MRI data were found in 67 bvFTD, 11 PPA, 11 FTD-ALS, 13 CBD, and 26 PSP patients. Of these EP symptoms were present in 19/67 bvFTD, 2/11 PPA, 1/11 FTD-ALS, 13/13 CBD and 25/26 PSP patients. Applicable FDG-PET data were found in 42 bvFTD, 6 PPA, 1 FTD-ALS, 7 CBD, and 8 PSP patients. Of these EP symptoms were present in 12/42 bvFTD, 2/6 PPA, 0/1 FTD-ALS, 7/7 CBD, and 8/8 PSP patients.

In the whole cohort, EP- patients were significantly younger compared to the EP + patients (65.8 vs. 69.1, *p* = 0.03) at the time of imaging. There was no significant difference in the gender distribution between the EP + and EP- groups. In the bvFTD group, there were no significant differences in gender distribution nor age between the EP + and EP- groups. The *C9orf72* repeat expansion status was available for 62/139 patients. In the EP + group, there were 7 repeat expansion carriers and 17 non-carriers. In the EP− group, there were 22 repeat expansion carriers and 16 non-carriers.

### Structural MRI in the EP + and EP− groups

First, we assessed the differences in cortical thickness between the EP + and EP− patients among all patients, and then in the bvFTD group separately. We found no differences among these patient groups.

Next, we focused on the volumes of the subcortical GM structures. Considering the whole patient group, EP + patients showed significantly lower volumes in the right putamen (*p* = 0.002*)*, bilateral globus pallidum (*p* = 0.002, *p* = 0.042; right (R), left (L)), right ventral diencephalon (*p* = 0.002), and brain stem (*p* < 0.001) when compared to the EP- patients. In further examination, the brain stem was segmented into subparts. All parts showed significantly lower volumes in the EP + group; medulla oblongata (*p* = 0.003), pons (*p* = 0.002), superior cerebellar peduncle (SCP) (*p* = 0.009), and midbrain (*p* = 0.001) (Tables 1 and 2). Other subcortical GM regions did not show any differences between the EP + and EP- groups.

**Table 1:** Comparison of cortical thickness (mm) and subcortical volumes (cm^3^) in volumetric MRI between patients with and without EP symptoms

		All patients			bvFTD only		
		EP + , mean (SD)	EP−, mean (SD)	*p*	EP + , mean (SD)	EP-, mean (SD)	*p*
Frontal	Left	1.42 (0.25)	1.43 (0.23)	0.852	1.38 (0.22)	1.41 (0.24)	0.668
	Right	1.42 (0.25)	1.44 (0.25)	0.995	1.37 (0.21)	1.41 (0.24)	0.556
Parietal	Left	1.31 (0.22)	1.31 (0.21)	0.776	1.29 (0.20)	1.29 (0.21)	0.981
	Right	1.29 (0.22)	1.32 (0.22)	0.733	1.27 (0.18)	1.29 (0.20)	0.748
Temporal	Left	1.61 (0.29)	1.61 (0.27)	0.810	1.59 (0.26)	1.58 (0.28)	0.886
	Right	1.61 (0.29)	1.62 (0.30)	0.827	1.60 (0.25)	1.58 (0.28)	0.754
Occipital	Left	1.09 (0.19)	1.10 (0.17)	0.957	1.07 (0.17)	1.09 (0.16)	0.670
	Right	1.11 (0.19)	1.13 (0.19)	0.815	1.08 (0.17)	1.11 (0.17)	0.640
Insula	Left	1.72 (0.31)	1.68 (0.27)	0.240	1.68 (0.26)	1.64 (0.27)	0.461
	Right Left	1.67 (0.31) 1.99 (0.39)	1.71 (0.31)	0.768 0.910	1.64 (0.24) 1.90 (0.37)	1.66 (0.29)	0.829 0.249
Caudate			1.99 (0.36)			2.02 (0.40)	
	Right	2.09 (0.39)	2.09 (0.44)	0.814	1.99 (0.41)	2.12 (0.50)	0.320
Putamen	Left	2.38 (0.39)	2.54 (0.43)	0.068	2.36 (0.47)	2.53 (0.44)	0.186
	Right	2.40 (0.41)	2.65 (0.41)	0.002	2.37 (0.48)	2.61 (0.44)	0.052
Pallidum	Left	1.11 (0.15)	1.17 (0.15)	0.042	1.10 (0.15)	1.18 (0.16)	0.086
	Right	1.08 (0.16)	1.18 (0.18)	0.002	1.06 (0.15)	1.16 (0.17)	0.027
Hippocampu	Left	2.26 (0.38)	2.21 (0.34)	0.125	2.27 (0.39)	2.21 (0.34)	0.468
	Right	2.27 (0.38)	2.27 (0.37)	0.588	2.27 (0.37)	2.22 (0.35)	0.541
Amygdala	Left	0.84 (0.18)	0.82 (0.16)	0.282	0.84 (0.20)	0.82 (0.17)	0.733
	Right	0.98 (0.18)	0.95 (0.20)	0.279	0.96 (0.16)	0.92 (0.17)	0.442
Accumbens	Left	0.20 (0.06)	0.21 (0.07)	0.676	0.19 (0.06)	0.19 (0.07)	0.857
	Right	0.24 (0.05)	0.25 (0.08)	0.515	0.22 (0.06)	0.24 (0.08)	0.339
Ventral DC	Left	2.23 (0.28)	2.35 (0.30)	0.081	2.18 (0.29)	2.35 (0.28)	0.028
	Right	2.17 (0.28)	2.36 (0.31)	0.002	2.13 (0.26)	2.35 (0.28)	0.004
Brainstem		15.20 (1.61)	16.28 (1.77)	< 0.001	14.78 (1.75)	16.45 (1.68)	0.001
	Medulla obl	2.65 (0.40)	2.86 (0.41)	0.003	2.59 (0.43)	2.90 (0.35)	0.004
	Pons	8.85 (1.00)	9.47 (1.12)	0.002	8.59 (1.13)	9.58 (1.14)	0.002
	SCP	0.15 (0.04)	0.17 (0.04)	0.009	0.14 (0.03)	0.17 (0.03)	0.001
	Midbrain	3.55 (0.38)	3.79 (0.36)	0.001	3.46 (0.38)	3.80 (0.36)	0.001
Thalamus	Left	4.05 (0.55)	4.08 (0.54)	0.734	3.98 (0.48)	4.02 (0.44)	0.777
	Right	3.95 (0.52)	4.00 (0.57)	0.990	3.92 (0.56)	3.93 (0.52)	0.952

Thicknesses and volumes presented are divided by TIV. The *p*-values stem from a regression model, also corrected for the age at scan

**Table 2:** Hypometabolic regions of EP + patients

All patients						
Hemisphere	Region (MNI space)	X	Y	Z	T	Cluster size*
L	Inferior temporal gyrus	− 48	− 6	− 38	6.36	5669
M	Medial Frontal Gyrus	2	34	− 16	5.72	6555
L	Postcentral Gyrus	− 42	− 24	40	5.32	206
R	Paracentral Lobule	16	− 36	54	5.05	731
L	Inferior Frontal Gyrus	− 54	16	18	4.55	390
R	Inferior Temporal Gyrus	52	− 6	− 34	3.94	209
bvFTD patients						
L	Inferior Temporal Gyrus	− 28	0	− 42	6.15	6408
R	Parahippocampal Gyrus	24	− 12	− 20	4.89	941
M	Medulla oblongata	6	− 42	− 46	4.25	254
L	Precentral Gyrus	− 40	− 26	38	4.11	225
M	Medial frontal gyrus	0	32	− 18	4.11	622
L	Insula	− 38	− 14	4	3.94	369
R	Lingual gyrus	24	− 76	− 18	3.70	108
R	Fusiform Gyrus	52	− 14	− 28	3.50	120
bvFTD EP + vs PSP/CBD						
M	Cerebellar lingula	2	− 46	− 18	4.91	667

(*): Hypometabolic regions of EP+ patients in the whole cohort, only bvFTD patients and bvFTD with EP symptoms compared to CBD and PSP patients (p < 0.001, uncorrected). R=Right, L=left, M=medial*Voxel size 2x2x2 mm. Coordinates are in MNI space

In the subgroup of only bvFTD patients, the results were partly similar and the EP + patients showed significantly lower volumes when compared to the EP- patients. The most prominent difference was observed in the brain stem (*p* = 0.001) and its subparts; medulla oblongata (*p* = 0.004), pons (*p* = 0.002), SCP (*p* = 0.001), and midbrain (*p* = 0.001), followed by right globus pallidum (*p* = 0.027) and bilateral ventral diencephalon (*p* = 0.004, *p* = 0.028; R,L). Other subcortical GM regions in the bvFTD subgroup did not show any difference between the EP + and EP- groups.

### ^18^FDG-PET in the EP + and EP− groups

**Fig. 1:**
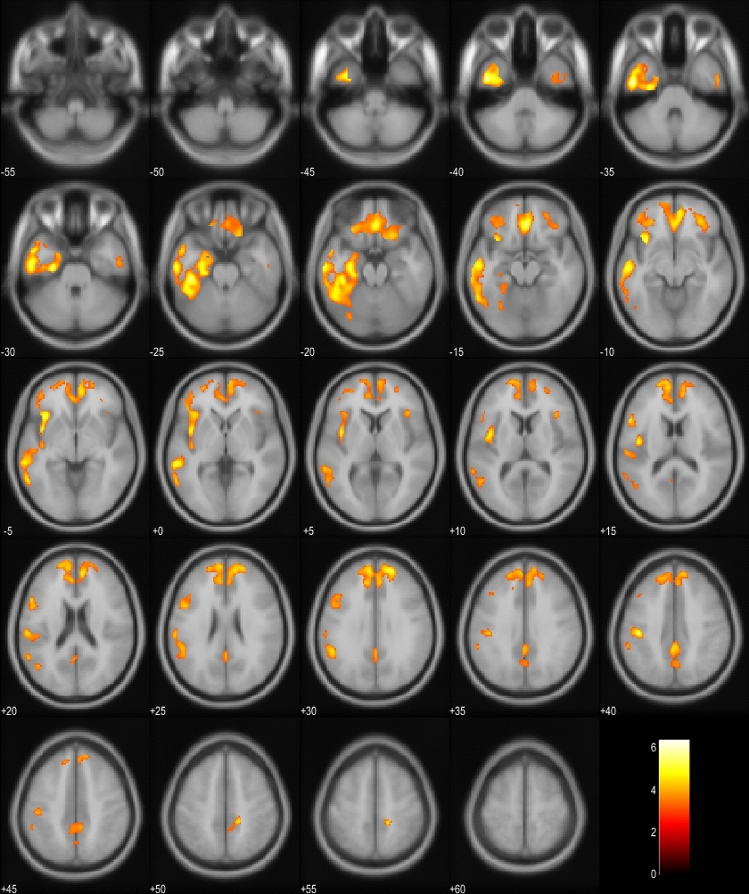
Hypometabolism in EP + patients compared to EP− patients in the whole cohort. Orange and yellow colors represent lower metabolism in the EP + group (*p* < 0.001, uncorrected). Images are presented in neurological orientation (*i.e.*, left hemisphere presented in left side of the picture)

^18^FDG-PET scans of the EP + patients were compared to the scans of the EP- patients in the whole cohort and also in the subgroup including only bvFTD patients (Fig. 1a, b). In the whole cohort, EP + patients showed significant hypometabolism predominantly in the left hemisphere in the temporal and medial frontal lobes. In the bvFTD subgroup, similar results were obtained in the EP + patients with hypometabolism detected mostly in the left temporal lobe. Also, the SCP and the frontal lobes showed hypometabolism. Comparison between EP + bvFTD patients and patients with PSP or CBD indicated that the bvFTD patients specifically showed hypometabolism in cerebellar lingula and SCP (Fig. 1c). No differences were observed in the cortical regions.

## Discussion

**Fig. 2:**
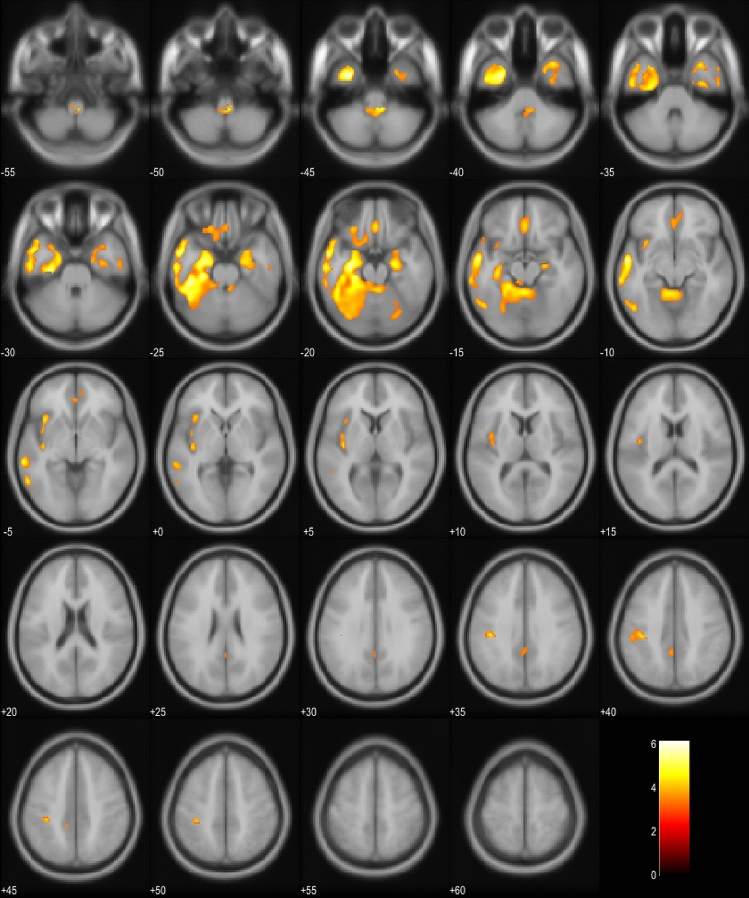
Hypometabolism in bvFTD EP + patients compared to bvFTD EP− patients. Orange and yellow colors represent lower metabolism in the EP + group (*p* < 0.001, uncorrected). Images are presented in neurological orientation (*i.e.*, left hemisphere presented in left side of the picture)

Here, we report an association between EP symptoms and brainstem atrophy in patients suffering from FTD spectrum disorders in general, and also specifically in the bvFTD patients. To the best of our knowledge, these are novel findings. EP symptoms in Parkinson’s disease patients are known to originate from atrophy of the basal ganglia in the midbrain (substantia nigra pars compacta). These patients are neuropathologically characterized mainly by α-synucleopathy. Also, PSP and CBD usually present EP symptoms, but in these disorders, the main underlying neuropathology is tauopathy, which is a neuropathology commonly detected in the FTD spectrum disorders [17]. Thus, different molecular neuropathologies could underlie the different topographical propagation of the neuropathological changes in the brain of these patients. In PSP, atrophy in the SCP and in the cerebrum at the level of the subthalamic nucleus (STN) is detected, whereas the SCP and STN are normal in Parkinson’s disease brain [18]. We also found hypometabolism in the SCP of the EP + bvFTD patients compared to EP- bvFTD patients. This finding could indicate that the EP symptoms of the FTD-related tauopathies originate from atrophy in the more caudal areas of the brainstem compared to patients with Parkinson’s disease. According to our data, the hypometabolism was asymmetrical between the two hemispheres. The asymmetry of neuropathology in *post-mortem* bvFTD brain has been reported in 2018 [19], but the underlying cause of this remains to be explored (Fig. 2).

**Fig. 3:**
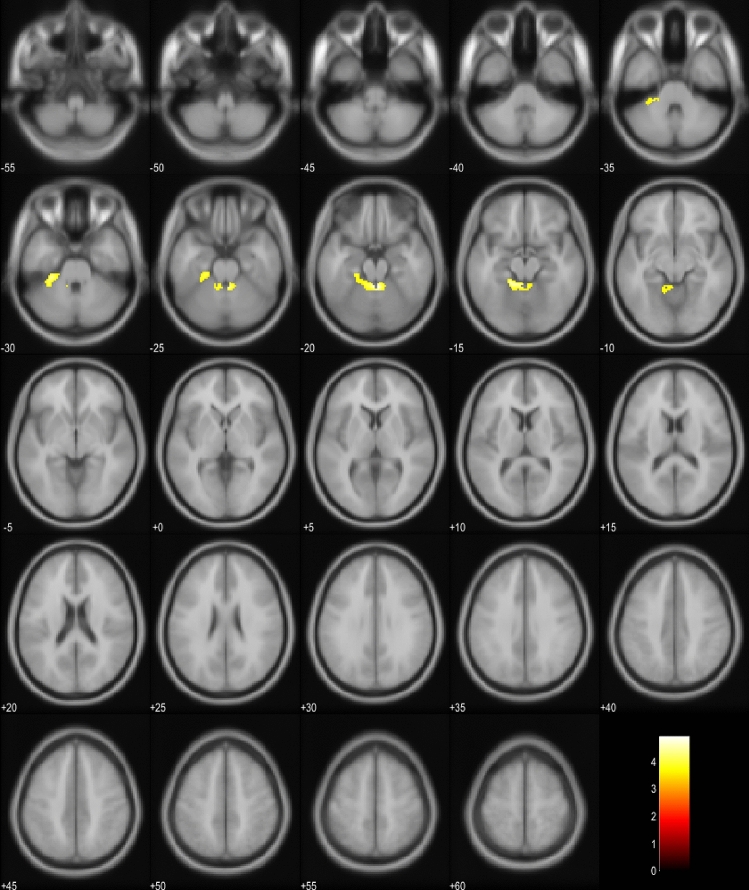
Hypometabolism in bvFTD patients with EP symptoms compared to PSP and CBD patients. Orange and yellow colors represent lower metabolism in the bvFTD group (*p* < 0.001, uncorrected). Images are presented in neurological orientation (*i.e*., left hemisphere presented in left side of the picture)

The previously reported neuroimaging findings in bvFTD patients are diverse and vary according to the disease-causing genetic mutation and underlying neuropathology. Our findings suggest that hypometabolism in the brain stem, SCP, and left temporal and frontal lobe as well as volume loss in the brain stem, globus pallidum, putamen, and SCP are linked to EP symptoms in bvFTD. The *C9orf72* repeat expansion has been reported to associate with atrophy of the more posterior and subcortical brain areas, including cerebellum, occipital and parietal cortex, thalamus, and the striatum, even at the presymptomatic phase [20–26]. Up to 14–48% of the patients carrying the *C9orf72* repeat expansion exhibit parkinsonism [27]. In Finland, a vast majority of familial bvFTD is caused by the *C9orf72* repeat expansion, which may at least partially explain our present findings. However, we observed prominent atrophy of the brain stem in the EP + bvFTD patients. This is not a common site of atrophy in the bvFTD patients, although it is often detected in CBD [28]. One explanation for this could be that the thalamus is one of the first structures to be affected by bvFTD [20, 21, 24, 29]. As thalamus is closely connected to the brain stem nuclei, its dysfunction could lead to Wallerian-like degeneration of the brain stem tracts and nuclei. The reason why only some but not all bvFTD patients develop brain stem atrophy remains to be elucidated (Fig. 3).

**Fig. 4:**
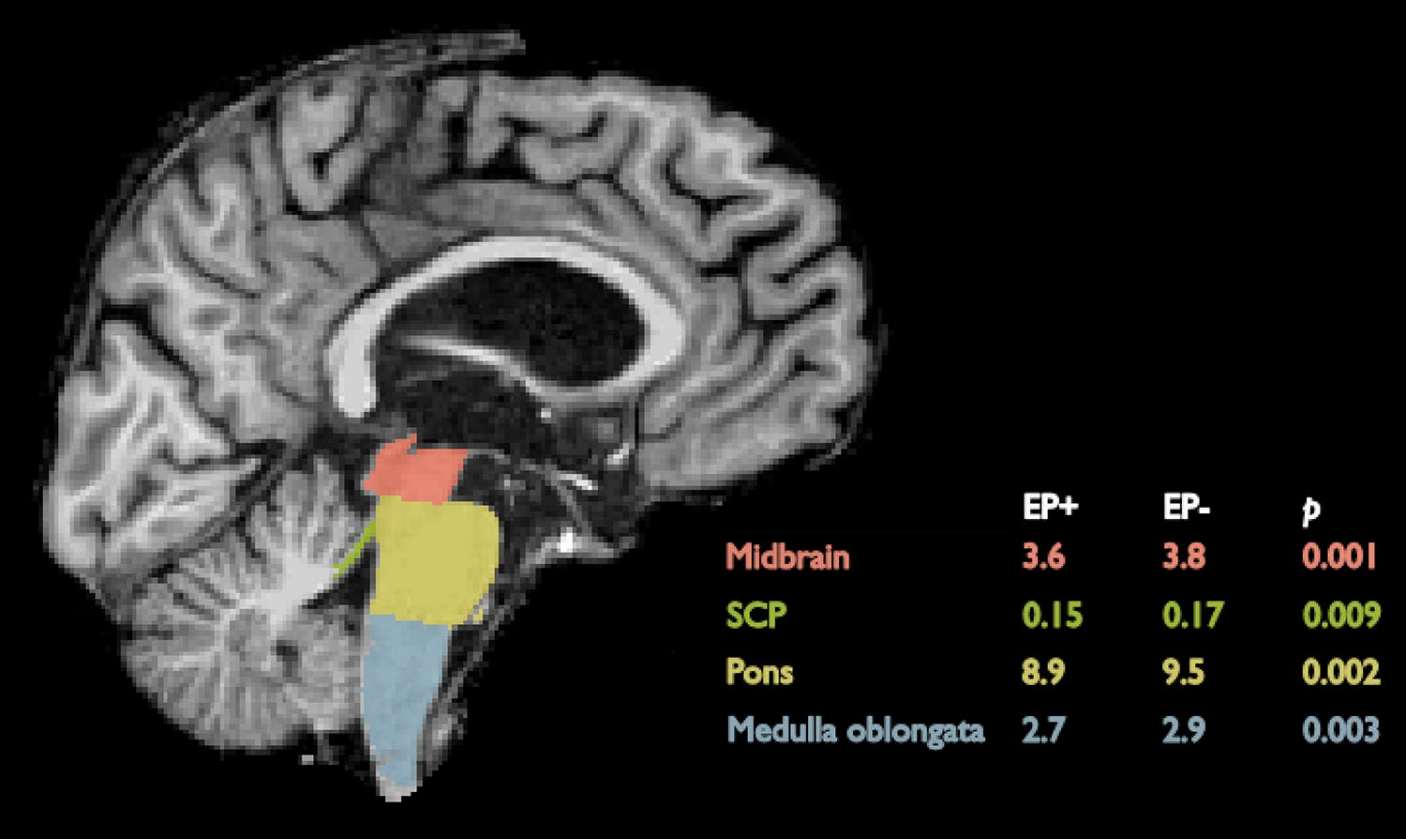
An illustration of brainstem structures and mean volumes (cm^3^) in the EP + and EP− groups. The structures are labeled on the brain of 58-year-old bvFTD patient with EP symptoms

The strengths of the present study include modern imaging methods and analyses with valid software. The diagnoses and the presence of EP symptoms were reviewed by a physician with special expertise in neurodegenerative diseases. However, the retrospective nature of this study limits drawing of the final conclusions. Moreover, the MRI scans were obtained at different time points with different scanners, varying sequence parameters, and field strengths, which may have affected the results. In the future studies, harmonization of imaging protocols should be preferred. Also, neuropathological confirmation of the patient groups combined with detailed genetic data would strengthen future studies (Fig. 4).

## Conclusion

Using automated image analysis tools, we discovered that bvFTD patients with EP symptoms have significant structural and metabolic differences in their brains compared to bvFTD patients without EP symptoms. Our findings could be utilized for earlier differentiation of such FTD spectrum patients from, e.g., Parkinson’s disease patients, enabling early and more accurate differential diagnosis of these rare neurodegenerative diseases. Reliable early diagnosis is a crucial step in the path toward the development of disease-modifying therapies and accurate treatment of patients with these devastating diseases.

## Abbreviations

bvFTD: Behavioral variant frontotemporal dementia
CBD: Corticobasal degeneration
DC: Diencephalon
EP: Extrapyramidal
FDG: Fluorodeoxyglucose
FTD: Frontotemporal dementia
FWE: Family-wise error
GM: Gray matter
L: Left
M: Medial
MNI: Montreal Neurological Institute
PPA: Primary progressive aphasia
PSP: Progressive supranuclear palsy
R: Right
STN: Subthalamic nucleus
SCP: Superior cerebellar peduncle
TDP-43: Transactive response DNA binding protein 43
TIV: Total intracranial volume
